# Platelet Delta (δ)-Storage Pool Deficiency: A Case Series and Review of the Literature

**DOI:** 10.3390/hematolrep15030041

**Published:** 2023-06-29

**Authors:** Amir F. Beirat, Sasmith R. Menakuru, Maitri Kalra

**Affiliations:** 1 Department of Internal Medicine, Indiana University School of Medicine, Muncie, IN 47303, USA; 2 Department of Hematology/Oncology, Indiana University School of Medicine, Muncie, IN 47303, USA

**Keywords:** platelets, platelet delta-storage pool disorder, platelet δ-storage pool deficiency, inherited platelet disorders

## Abstract

Hereditary platelet delta (δ)-storage pool deficiency is a rare condition in which there are fewer dense granules in platelets disrupting primary hemostasis. It can cause a mild–moderate bleeding tendency with normal coagulation studies; hence, it is an underdiagnosed diagnostic challenge. The authors present three patients with hereditary platelet delta (δ)-storage pool deficiency who had heavy menstrual bleeding, excessive bleeding following surgery, mucocutaneous bleeding, and a bleeding score greater than or equal to 6. These cases reveal the susceptibility of underdiagnosing platelet disorders and the significance of utilizing a bleeding assessment tool to help guide further workup with transmission electron microscopy to visualize the fewer dense granules in platelets. Although bleeding is typically moderate, it can be severe in certain scenarios, like after mucosal surgeries, and can lead to death, highlighting the importance of the condition’s recognition and prophylactic treatment.

## 1. Introduction

Platelets are one of the initial cells responsible for stopping bleeding, mainly via primary hemostasis, which involves adhesion to sites of vascular injury and the aggregation of platelets [1]. The platelet secretion of various proteins, which come from one of two platelet granules, alpha and dense granules, facilitate the process of primary hemostasis. Von Willebrand factor (vWF) and fibrinogen are among the many proteins found in alpha granules. In contrast, dense granules store and release adenosine diphosphate (ADP), adenosine triphosphate (ATP), ionized calcium, histamine, and serotonin. As a result, more platelets are recruited, and their activation is augmented [2]. It is well established that bleeding can result from platelet disorders, whether acquired or congenital.

Platelet delta (δ)-storage pool disorders can either be in the form of a syndrome, such as Chediak–Higashi disease (CHD), Hermansky–Pudlak syndromes (HPS), Griscelli syndrome, or a non-syndromic form. Disorders are characterized by either qualitative or quantitative defects. Non-syndromic forms with qualitative defects can be from the failure of granular secretion in response to collagen as well as other agonists. On the other hand, quantitative defects can be from a decrease in the number or content of the dense granules, such as hereditary δ-storage pool deficiency, a rare and underdiagnosed heterogeneous group of platelet disorders. Platelets usually contain between three and eight dense granules. A lower number can manifest with easy bruising, mucosal bleeding, or excessive bleeding following surgery. Bleeding is typically mild to moderate, leading to a missed diagnosis. Rarely do some patients have severe bleeding that can lead to death [3,4]. The use of a standardized bleeding assessment tool (BAT), which considers prior bleeding history to calculate a bleeding score (BS), can aid in the evaluation of suspected bleeding disorders [5]. We present three cases of hereditary δ-storage pool deficiency with excessive post-surgical bleeding, heavy menstrual bleeding, and mucocutaneous bleeding. What is interesting about these three patients is that they belong to the same family. Here, we delve into the question of other possible familial/genetic associations of some non-syndromic forms of delta-storage pool disorders. These cases did not have any clinical features of Chediak–Higashi disease, Hermansky–Pudlak syndrome, or Griscelli syndrome. We also discuss the diagnosis and management of these patients in hopes of raising awareness on the topic.

## 2. Case Presentations

**Case 1**

We present a 28-year-old obese white female with a medical history significant for vitamin B12 deficiency, excessive post-partum bleeding, and heavy menstrual bleeding. She was referred to the hematology clinic to evaluate her bleeding tendency before a tonsillectomy and adenoidectomy. Her menstrual history consisted of seven days of heavy and profuse bleeding that required her to use super tampons and pads simultaneously. Her obstetrics history was significant for two vaginal deliveries, both followed by post-partum hemorrhage requiring the use of oxytocin to control the bleeding. In contrast, she had an appendectomy and ovarian torsion repair with no bleeding complications. She also previously had an episode of hematuria, which resolved, but no history of gastrointestinal bleeding.

Family history was significant as her brother had a tonsillectomy as a child and had a delayed hemorrhage on post-operative day 1 requiring cauterization. Similarly, at the age of 2 years old, her son had a tonsillectomy causing a delayed hemorrhage on post-operative day 1 leading to re-exploration surgery. Her grandmother and her mother also had issues with epistaxis throughout their lives. Her mother has menorrhagia and receives IV iron for iron deficiency anemia. However, she had no history of significant epistaxis, easy bruising, or petechiae. She had a BS of 6. Other medical history included asthma and tobacco use. For her social history, she was not a smoker, did not drink alcohol or take illicit substances, and did not take unusual over-the-counter medications. She was not of Mediterranean or Italian descent.

The physical examination did not show hypopigmentation of skin or hair and was otherwise unremarkable. The initial laboratory work-up (see Table 1) included a complete blood count (CBC), prothrombin time (PT) with international normalized ratio (INR), activated partial thromboplastin time (aPTT), and thrombin clotting time (TCT). Given normal results, further testing with fibrinogen; factor VIII, XI, and XIII assays; a vWF screen; and a platelet function analyzer was performed (see Table 1). The platelet function analyzer showed a mildly decreased platelet response only to collagen. Previous labs did not yet result in a diagnosis. Eventually, her platelets were evaluated by transmission electron microscopy (TEM) (see Table 2) and showed an average of 2.73 dense granules per platelet (reference range: 3.68–6.24). Given her history of bleeding and no signs or other symptoms to suggest a genetic syndrome, she was diagnosed with platelet δ-storage pool deficiency.

Two days before her surgery, she was given two units of platelets and had an uncomplicated procedure. In the case of bleeding, it was planned that she would receive aminocaproic acid at 50–60 mg/kg every four hours after surgery if she had bleeding complications, but it was not required. She was later involved in a minor motor vehicle accident requiring hip arthroplasty and received prophylactic intravenous desmopressin, preventing bleeding complications.

She was referred for gastroenterology and pulmonary consultations and was not found to have any evidence of pulmonary fibrosis or colitis, which could be concerning for HPS or CHD. Her two children were evaluated by their pediatrician and found to have delta granule deficiency. Furthermore, in order to control her heavy menstrual bleeding, a combined oral contraceptive pill was started. Plans for future surgeries consisted of giving desmopressin (DDVAP) once intravenously (IV) at 0.3 mcg/kg 30–60 min prior to a procedure or tranexamic acid 1 gm IV at 100 mg/min for 30 min prior to a procedure. She is currently doing well and follows up at the hematology clinic before any surgical procedure.

**Case 2**

We present a 40-year-old morbidly obese white female with a history significant for easy bruising, menorrhagia complicated by iron-deficiency anemia and treated with IV iron transfusions, and tracheal stenosis requiring dilatation every two years without bleeding complications. She was being evaluated for platelet δ-storage pool deficiency due to the diagnosis of her stepsister from a different father (case 1).Her history did not include recurrent epistaxis, but she had a prior episode of epistaxis that lasted forty minutes and required a nasal tampon to control the bleeding. She did not have any history of blood in her stools, dark stools, hematemesis, petechiae, or bleeding gums. Her menstrual cycle, albeit irregular due to her being peri-menopausal, consisted of heavy bleeding one to two times a year lasting several weeks, requiring changing pads five to six times a day. Before being peri-menopausal, her cycles also needed daily pad changes five to six times. Obstetric history included one vaginal delivery with no bleeding complications. In the past, she had a tonsillectomy with no bleeding complications and did not undergo other surgeries. The calculated BS was 6. Other medical history included obstructive sleep apnea, hypertension, and hypothyroidism. Social history was unremarkable and like that of case 1. The physical examination was unremarkable. The laboratory work-up included a CBC, PT with INR, aPTT, TCT (see Table 1), and a platelet evaluation with TEM (see Table 2), which showed an average of 1.8 dense granules per platelet. She was then diagnosed with platelet δ-storage pool deficiency and is currently doing well, being managed similarly to case 1.

**Case 3**

Lastly, we present a case of a 48-year-old obese white female with a history significant for uterine fibroids and heavy menstrual bleeding complicated by iron-deficiency anemia treated with IV iron transfusions. Just as in the second case, she was being evaluated for platelet δ-storage pool deficiency after the diagnosis was made in her daughter (case 1). She is menopausal, but her prior menstrual history consisted of cycles every four weeks lasting five to seven days with heavy bleeding and large clots requiring pad changes every two hours. She did not have recurrent epistaxis but an episode of epistaxis with a large amount of blood and clots that required cauterization to control the bleeding. Obstetric history was relevant for a delayed postpartum hemorrhage after her first vaginal delivery, and she required dilatation and curettage to stop the bleeding. Also, unfortunately, she had a spontaneous abortion complicated by heavy bleeding.

She previously underwent a cholecystectomy with no bleeding complications. She had no blood in her stools or black-colored stools, hematemesis, bleeding gums, easy bruising, or petechiae. Her BS was 10. Her medical history included hypothyroidism and hypertension. Social history was also like that of case 1. The physical examination was unremarkable. Given her family history, she only had a CBC (see Table 1) and platelet evaluation with TEM (see Table 2), which revealed 1.66 dense granules per platelet, leading to the diagnosis of platelet δ-storage pool deficiency. She is also currently doing well. Given her post-menopausal state, she is being managed conservatively and has been directed to contact the hematology office prior to any procedures with the plan to treat with DDAVP prophylactically prior to procedures.

## 3. Discussion

Platelet δ-storage pool deficiency is a rare cause of bleeding diathesis. Platelet dis-orders are associated with primary hemostasis [1]. Thus, bleeding presentation is associated with mucocutaneous bleeding, including epistaxis, petechiae, heavy menstrual bleeding, or prolonged and excessive bleeding after surgery. In contrast, bleeding into deep tissues and large ecchymosis are characteristic of disorders related to secondary hemostasis [6]. Overlap in symptoms can occur, highlighting the importance of a detailed history, specifically a bleeding history and physical examination. Also, underlying medical conditions and medications, including over-the-counter medications, are crucial to rule out acquired causes [6]. We present three cases of hereditary platelet δ-storage pool deficiency, all with a history of heavy menstrual bleeding and post-surgical bleeding requiring intervention, either surgically or with medication, and a family history significant for mucocutaneous and excessive post-surgical bleeding.

All patients in the reported cases refused to be evaluated for a genetic disorder by genetic testing. However, not performing this evaluation raises a crucial question of the possible involvement of certain genetic factors in delta granule deficiency presenting in a non-syndromic manner. We are aware of the genetic associations in HPS, in which we find genetic mutations in the HPS1 and HPS4 genes [7]. Similarly, we know that Chediak–Higashi syndrome results from mutations in the LYST gene, which encodes a cytosolic protein that is probably involved in granule trafficking and/or fusion, and the clinical manifestations of Griscelli syndrome result from mutations in the RAB27A gene [8]. Most non-syndromic forms of platelet δ-storage disorders were noted to result from a deficit in nucleotides, particularly ADP. It is suspected that the nucleotide transporter MRP4, which is encoded in the ABCD4 gene, may be implicated in the pathology, but mutations are yet to be identified [3]. Although all patients were female and obese, the ABCD4 gene is in chromosome 14 and not the X chromosome. Further research is warranted into the involvement of other genes in delta granule deficiency. As a clinician, it is essential to obtain a good family history and screen for bleeding disorders in family members of patients diagnosed with non-syndromic delta granule deficiency. The possibility of simultaneously exploring several genes using next-generation sequencing techniques (gene-panel, whole-exome, and whole-genome sequencing) should make it possible to identify new genes involved in these deficits in the coming years. We present these cases to raise awareness among physicians about the approach to patients with inherited platelet disorders.

The International Society on Thrombosis and Haemostasis (ISTH) developed the BAT to consider a person’s bleeding history and results in a bleeding score (BS), which is abnormal if greater than 4 and 6 in adult males and females, respectively [5,9,10]. An abnormal BS increases the likelihood of a bleeding disorder and warrants a laboratory workup. Initially, this includes a CBC and coagulation studies with PT and aPTT. A platelet function analyzer (PFA) can also be performed and is affected by low platelet count, platelet dysfunction, vWF activity, and hematocrit. This test has mostly replaced the use of bleeding time but has low sensitivity and is of questionable benefit. Depending on the diagnostic yield of the previously stated studies, a more extensive workup is conducted based on whether a disorder in primary or secondary hemostasis is suspected [11].

Our patient in case 1 had normal CBC, PT, aPTT, and PFA values. Following a step-by-step approach [12], she had a peripheral smear with no visible platelet abnormalities and a light transmission aggregometry (LTA) assessing platelet aggregation, which showed a mildly decreased platelet response only to collagen. Eventually, platelets were evaluated with transmission electron microscopy (TEM) and demonstrated fewer dense granules, leading to the diagnosis of platelet δ-storage pool deficiency. In support, as LTA can show a false negative, it is reasonable to evaluate with TEM in patients with a BS > 6, given the exclusion of von Willebrand disease (vWD) [13,14]. Interestingly, Gresele et al. demonstrated that a BS > 6 increased the likelihood of bleeding events in inherited platelet disorders and that a higher baseline BS was associated with more bleeding events, which can help determine the need for treatment [15].

Management initially involves avoiding medications that alter platelet physiology and increase the likelihood of bleeding, such as aspirin, other anti-platelet drugs, or selective serotonin reuptake inhibitors. Secondly, the mainstay of treatment is managing the consequences of platelet disorders, using, for example, oral contraceptive medication or iron supplementation in cases of heavy menstrual bleeding and iron deficiency anemia, respectively [4,14,15]. Furthermore, anti-fibrinolytic agents (tranexamic acid or aminocaproic acid) and desmopressin are used prophylactically before a procedure or surgery to prevent/control bleeding. Platelet transfusion should be limited to the failure of the former two to control bleeding, and it is not recommended for prophylaxis [3,16,17]. For case 1, prophylactic platelet transfusion was performed for her first surgery, and desmopressin was administered for a second different surgery, preventing bleeding complications with both. In support, Orsini et al. demonstrated that desmopressin was the most effective for prophylaxis. On the other hand, Lundy et al. compared the rates of major bleeding between prophylactic platelet products and non-platelet products and failed to find any significant differences [18].

## 4. Conclusions

In conclusion, we report three rare and likely late-diagnosed cases of hereditary platelet δ-storage pool deficiency with symptoms of excessive bleeding during menstruation and following surgery. Our cases highlight the possible involvement of genetic factors in delta granule deficiency presenting in a non-syndromic manner and the importance of utilizing bleeding assessment tools in patients with suspected inherited platelet disorders.

## Figures and Tables

**Table 1:** Baseline lab values.

Lab Variables	Case 1	Case 2	Case 3	Reference Range
WBC	7.1	6.8	6.9	3.6–10.6 k/cumm
RBC	4.62	4.61	4.62	3.71–5.17 million/cumm
Platelets	438	310	339	150–450 k/cumm
MPV	6.8	8.4	8.1	7–12 fL
PT/INR	12.7/1.13	13.2/1.18	-	9.3–13.5 s/0.75–1.26
aPTT	35.1	35	-	23.4–38 s
TCT	14.2	-	-	10.7–14.6 s
Fibrinogen	336	-	-	170–399 mg/dL
Factor VIII assay	187%	-	-	61–155%
Factor XI assay	125%	-	-	77–162%
Factor XIII assay	115%	-	-	69–143%
vWF activity screen	107%	-	-	43–195%
vWF Ag	109%	-	-	44–167%
vWF collagen binding assay	77%	-	-	40–183%

Cases 2 and 3 did not have complete work-ups as it was unnecessary for making the diagnosis. Abbreviations: WBC: white blood cells. RBC: red blood cells. MPV: mean platelet volume. PT: prothrombin time. aPTT: activated partial thromboplastin time. TCT: thrombin clotting time. vWF: von Willebrand factor.

**Table 2:** Platelet evaluation with TEM.

Number of Dense Granules Per Platelet *	Case 1	Case 2	Case 3
0	22%	32%	41%
1–2	36%	37%	34%
3–4	22%	18%	15%
5–6	9%	9%	6%
7–8	7%	3%	2%
9–10	2%	1%	1%
>10	2%	0%	1%
Range	0–26	0–9	0–15
Mean	2.73	1.80	1.66

* Platelets were examined ultra-structurally using the whole-mount technique. Reference range of dense granules per platelets was 3.68–6.24, which was established using non-bleeding adult control patient populations. A total of 300 individual platelets were examined for each patient.

## Data Availability

Not applicable.

## References

[1] Jackson S.P. (2007). The growing complexity of platelet aggregation. Blood.

[2] Gremmel T., Frelinger A.L., Michelson A.D. (2016). Platelet Physiology. Semin. Thromb. Hemost..

[3] Dupuis A., Bordet J.-C., Eckly A., Gachet C. (2020). Platelet δ-Storage Pool Disease: An Update. J. Clin. Med..

[4] Masliah-Planchon J., Darnige L., Bellucci S. (2013). Molecular determinants of platelet delta storage pool deficiencies: An update. Br. J. Haematol..

[5] Rodeghiero F., Tosetto A., Abshire T.C., Arnold D.M., Coller B.S., James P.D., Neunert C.E., Lillicrap D. (2010). ISTH/SSC bleeding assessment tool: A standardized questionnaire and a proposal for a new bleeding score for inherited bleeding disorders. J. Thromb. Haemost..

[6] Girolami A., Luzzatto G., Varvarikis C., Pellati D., Sartori R., Girolami B. (2005). Main clinical manifestations of a bleeding diathesis: An often disregarded aspect of medical and surgical history taking. Haemophilia.

[7] Martina J.A., Moriyama K., Bonifacino J.S. (2003). BLOC-3, a Protein Complex Containing the Hermansky-Pudlak Syndrome Gene Products HPS1 and HPS4. J. Biol. Chem..

[8] Wilson S.M., Yip R., Swing D.A., O’Sullivan T.N., Zhang Y., Novak E.K., Swank R.T., Russell L.B., Copeland N.G., Jenkins N.A. (2000). A mutation in *Rab27a* causes the vesicle transport defects observed in *ashen* mice. Proc. Natl. Acad. Sci. USA.

[9] Kuiper P. ISTH-SSC Bleeding Assessment Tool. bleedingscore.certe.nl.

[10] Elbatarny M., Mollah S., Grabell J., Bae S., Deforest M., Tuttle A., Hopman W., Clark D.S., Mauer A.C., Bowman M. (2014). Normal range of bleeding scores for the ISTH-BAT: Adult and pediatric data from the merging project. Off. J. World Fed. Hemoph..

[11] Boender J., Kruip M.J., Leebeek F.W. (2016). A diagnostic approach to mild bleeding disorders. J. Thromb. Haemost..

[12] Gresele P., Harrison P., Gachet C., Hayward C., Kenny D., Mezzano D., Mumford A., Nugent D., Nurden A., Cattaneo M. (2015). Diagnosis of inherited platelet function disorders: Guidance from the SSC of the ISTH. J. Thromb. Haemost..

[13] Gunning W.T., Yoxtheimer L., Smith M.R. (2021). Platelet Aggregation Assays Do Not Reliably Diagnose Platelet Delta Granule Storage Pool Deficiency. J. Hematol..

[14] Gresele P., Orsini S., Noris P., Falcinelli E., Alessi M.C., Bury L., Borhany M., Santoro C., Glembotsky A.C., Cid A.R. (2020). Validation of the ISTH/SSC bleeding assessment tool for inherited platelet disorders: A communication from the Platelet Physiology SSC. J. Thromb. Haemost..

[15] Gresele P., Falcinelli E., Bury L., Pecci A., Alessi M., Borhany M., Heller P.G., Santoro C., Cid A.R., Orsini S. (2021). The ISTH bleeding assessment tool as predictor of bleeding events in inherited platelet disorders: Communication from the ISTH SSC Subcommittee on Platelet Physiology. J. Thromb. Haemost..

[16] Bolton-Maggs P.H.B., Chalmers E.A., Collins P.W., Harrison P., Kitchen S., Liesner R.J., Minford A., Mumford A.D., Parapia L.A., Perry D.J. (2006). A review of inherited platelet disorders with guidelines for their management on behalf of the UKHCDO. Br. J. Haematol..

[17] Orsini S., Noris P., Bury L., Heller P.G., Santoro C., Kadir R.A., Butta N.C., Falcinelli E., Cid A.R., Fabris F. (2017). Bleeding risk of surgery and its prevention in patients with inherited platelet disorders. Haematologica.

[18] Lundy K.A., Rabatin A., Davidson E.R., Li J., Snider M.J., Kraut E.H. (2022). Experience with Pre-procedural Hemostatic Medications versus Platelet Transfusion in Patients with Platelet Storage Pool Deficiency. J. Pharm. Pract..

